# Stability and Compatibility Studies of Levothyroxine Sodium in Solid Binary Systems—Instrumental Screening

**DOI:** 10.3390/pharmaceutics12010058

**Published:** 2020-01-10

**Authors:** Ionuț Ledeți, Mirabela Romanescu, Denisa Cîrcioban, Adriana Ledeți, Gabriela Vlase, Titus Vlase, Oana Suciu, Marius Murariu, Sorin Olariu, Petru Matusz, Valentina Buda, Doina Piciu

**Affiliations:** 1 Department of Pharmacy I, Faculty of Pharmacy, Victor Babes University of Medicine and Pharmacy, 300011 Timișoara, Romania; ionut.ledeti@umft.ro (I.L.); mirabela_romanescu@yahoo.com (M.R.); circioban.denisa@umft.ro (D.C.); 2 Research Centre for Thermal Analysis in Environmental Problems, West University of Timișoara, 300115 Timișoara, Romania; 3 Department of Medicine XIV, Faculty of Medicine, Victor Babes University of Medicine and Pharmacy, 300011 Timișoara, Romania; 4 Department of Medicine X, Faculty of Medicine, Victor Babes University of Medicine and Pharmacy, 300011 Timișoara, Romania; olariu.sorin@umft.ro; 5 Department of Medicine I, Faculty of Medicine, Victor Babes University of Medicine and Pharmacy, 300011 Timișoara, Romania; matusz@umft.ro; 6 Department of Pharmacy II, Faculty of Pharmacy, Victor Babes University of Medicine and Pharmacy, 300011 Timișoara, Romania; buda.valentina@umft.ro; 7 Department of Nuclear Medicine, Prof. Dr. Ion Chiricuță Institute of Oncology, 400015 Cluj-Napoca, Romania; doina.piciu@umfcluj.ro; 8 Department of Oncology, Faculty of Medicine, Iuliu Hațieganu University of Medicine and Pharmacy, 400012 Cluj-Napoca, Romania

**Keywords:** levothyroxine sodium, excipient, preformulation study, instrumental analysis

## Abstract

The influence of excipients on the stability of sodium levothyroxine pentahydrate (LTSS) under ambient conditions and thermal stress was evaluated. Since LTSS is a synthetic hormone with a narrow therapeutic index, the interactions of LTSS with excipients can lead to a drastic diminution of therapeutic activity. Ten commonly used pharmaceutical excipients with different roles in solid formulations were chosen as components for binary mixtures containing LTSS, namely, starch, anhydrous lactose, D-mannitol, D-sorbitol, gelatin, calcium lactate pentahydrate, magnesium stearate, methyl 2-hydroxyethyl cellulose (Tylose), colloidal SiO_2_ (Aerosil) and talc. As investigational tools, universal attenuated total reflectance- Fourier transform infrared spectroscopy UATR-FTIR spectroscopy and thermal analysis were chosen and used as follows: UATR-FTIR spectra were drawn up for samples kept under ambient conditions, while thermoanalytical tools (TG/DTG/HF data) were chosen to evaluate the inducing of interactions during thermal stress. The corroboration of instrumental results led to the conclusion that LTSS is incompatible with lactose, mannitol and sorbitol, and these excipients should not be considered in the development of new generic solid formulations.

## 1. Introduction

Levothyroxine sodium pentahydrate (LTSS) is the sodium salt of the levo-isomer of thyroxine, an active physiological substance found in the thyroid gland [1,2]. Endogenous production of thyroxine ([T4] 3,5,3′,5′ tetraiodothyronine) and triiodothyronine ([T3] 3,5,3′ triiodothyronine) is the primary function of the human thyroid [3]. These hormones are crucial in the regulation of many metabolic processes and are vital for normal growth and development. They are also involved in calorigenic, cardiovascular and metabolic effects. The hormones exert their effects presumably by activating gene transcription of messenger RNA and proteins. To do so, they enter the cell nucleus and bind to DNA-bound thyroid receptors, which regulate gene transcription [4].

A synthetic version of thyroxin is primarily used in the treatment of hypothyroidism and as a thyroid-stimulating hormone suppressant in the treatment of various types of euthyroid goiters [2].

Although thyroxine (T4) monotherapy is widely accepted as the core treatment for hypothyroidism, other therapies such as desiccated thyroid, triiodothyronine T3 monotherapy and combinations of T4 and T3 are also available [4].

**Figure 1:**
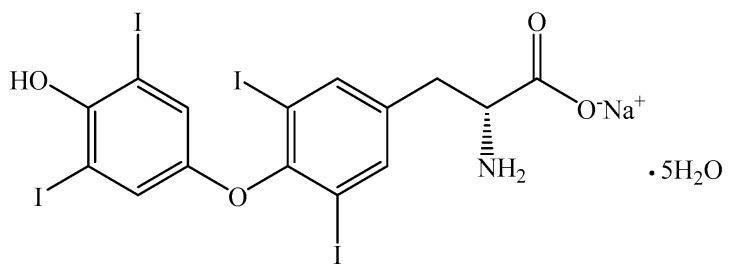
Structural formula of levothyroxine sodium salt pentahydrate (LTSS).

Levothyroxine is also used to treat euthyroid goiters including thyroid nodules, subacute or chronic lymphocytic thyroiditis, multinodular goiter or for thyroid cancer patients who have undergone thyroidectomy, and as an adjunct to surgery and radioiodine therapy [4]. Structural formula of levothyroxine sodium salt pentahydrate (abbreviated LTSS) is presented in Figure 1.

Commercial levothyroxine oral formulations available in North America and Europe include powders for intravenous solutions, tablets, soft gel capsules and oral solutions [5]. Many studies have been published describing comparisons of the pharmacokinetic properties of various levothyroxine formulations and various approaches have been employed since bioequivalence assessments of levothyroxine are complicated by baseline levels, feedback mechanisms and by the fact that levothyroxine is considered by some to be a “narrow therapeutic index” (NTI) drug [6,7].

The development of new solid formulations with levothyroxine content is of greatly importance, as of consequence of NTI of this hormone, since small variations in the quantity of API in each formulation have been associated with considerable variations in thyroid function in people with hypothyroidism [6]. Also, it is well known that usually formulations including hormones contain small quantities of active pharmaceutical ingredients (APIs), mostly in the order of micrograms, so if interactions occur between API and excipients, they will drastically affect the biodisponibility and, as a consequence, the therapeutic effect. Taking this into account, there are more stringent regulations regarding the API content in these formulations, in comparison to other drugs [6].

Several recent publications investigated the stability of levothyroxine, and the development of new formulations with better stability [6,8,9,10,11,12,13,14,15,16,17,18,19]. The effect of excipients on the stability of levothyroxine sodium pentahydrate tablets was studied by Gupta et al. and reported in 1990 [20] and aimed towards the transformations that took place in a solid formulation containing LTSS along with excipients that act as a catalyst to decomposition of API, leading to a shorter shelf-life of the product than the one mentioned by the manufacturer. Since good manufacturing practice requires that compatibility and stability studies must be conducted in the preformulation stage to assure the quality of the product, Patel et al. [21] reported the effect of various excipients (such as lactose anhydrous, starch, microcrystalline cellulose, dibasic calcium phosphate and mannitol) on the stability of LTSS in aqueous slurries and in formulated tablets. The study of Patel [21] revealed that tablets manufactured with dibasic calcium phosphate and basic pH modifier (like sodium carbonate, sodium bicarbonate, or magnesium oxide) led to improved stability of levothyroxine sodium pentahydrate tablets.

D. Concordet et al. reported, in March 2017, a new formulation of Levothyrox^®^ was licensed in France, as requested by the French authorities, aiming towards improving the stability issues of the old formulation; even if the bioequivalency of the two formulations was confirmed, adverse drug reactions were reported for several thousands of patients after administrations of the new formulation [8]. In 2019, Lipp and Hostalek [6] developed a new formulation of levothyroxine which is more stable, with a shelf life of three years, independent of geographic climate zones. Shah et al. studied the solid-state changes that take place during LTSS pentahydrate dehydration and its correlation to chemical instability, since tablets containing this API were one of the most highly recalled products due to potency and dissolution failures on stability [12]. Also, since LTSS has a NTI, the splitting of tablet can lead at under or excessive dosage, since distribution of API in solid formulation can be heterogeneous and dependent on type of binder and the process used in the manufacture of tablets [22].

Following all these presented considerations, the compatibility of a NTI drug such as LTSS and pharmaceutical excipients is extremely critical in the formulation of a quality drug product. The formulation of a stable and effective dosage form requires careful selection of excipients used to facilitate administration, promote consistent release and bioavailability of the drug, and to promote the active moiety from the environment. Although often regarded as inert, excipients can in fact readily interact with drugs [2].

Levothyroxine has a complex stability profile and is sensitive to various environmental factors such as light, air oxygen, and humidity [2,16,17]. It was suggested that the possible reasons for degradation of levothyroxine were the use of different excipients, the pH of the formulation, and the compression force used during tablet compaction. Therefore, a careful selection of excipients could be helpful to prevent potency loss over the shelf life of the tablets of levothyroxine [12].

Considering all of the above, further preformulation studies regarding levothyroxine compatibility need to be done in order to optimize its stability and bioavailability. There were selected a number of solid excipients, having different properties and playing various roles into the final formulation. The following excipients were included: starch, anhydrous lactose, D-mannitol, D-sorbitol, gelatin, calcium lactate pentahydrate, magnesium stearate, methyl 2-hydroxyethyl cellulose (Tylose), colloidal SiO_2_ (Aerosil) and talc, while, as instrumental techniques, UATR-FTIR spectroscopy and thermoanalytical tools were chosen, by a methodology previously reported in the literature [23,24,25,26,27,28,29,30,31].

## 2. Materials and Methods

### 2.1. Reagents

Levothyroxine sodium salt hydrate (LTSS, purity >99%, Acros Organics, Geel, Belgium, lot A0141090, CAS 25416-65-3) is a commercial product and was used without a priori purification. The producer of LTSS mentions solely that the product is hydrated, without providing the exact water content. This content was previously established in our previous study [15] and confirmed by the current obtained experimental data, as shown in Section 3.2.

As excipients, starch (StarCap 1500, Colorcon, Harleysville, USA), lactose anhydrous (Sigma Aldrich, Darmstadt, Germany), D-mannitol (Sigma Aldrich, Darmstadt, Germany), D-sorbitol (Fisher Scientific, Loughborough, UK), gelatin (Merck, Darmstadt, Germany), calcium lactate pentahydrate (CaLact, Sigma Aldrich, Darmstadt, Germany), magnesium stearate (Mosselman, Mons, Belgium), methyl 2-hydroxyethyl cellulose (Tylose, Sigma Aldrich, Darmstadt, Germany), colloidal SiO_2_ (Aerosil 200, Evonik Degussa, Essen, Germany) and talc (Imerys Talc, Malanaggio, Italy) were selected. All the excipients were used as received, without further purification. For all compounds, the producers declared that they are suitable for use as excipients.

### 2.2. Preparation of Samples

The binary mixtures were prepared by trituration of LTSS and each excipient in 1:1 (mass/mass) proportion, in an agate mortar with pestle for approximately 5 min. The methodology of preparing 1:1 ratio (mass/mass) binary mixtures aims to accentuate the drug–excipient interaction, irrespective of the exact concentration of the API in a real formulation. Immediately after preparation, samples consisting in binary mixtures were sealed in hermetically closed vials and kept under ambient conditions (20 ± 2 °C), in absence of light. As a standard of LTSS, the same amount of sample was triturated in identical conditions and sealed in a vial without adding any excipient.

### 2.3. Spectroscopic Investigations

UATR-FTIR spectra were recorded on Perkin Elmer SPECTRUM 100 device (Perkin-Elmer Applied Biosystems, Foster City, CA, USA), without a priori preparation of the sample. The data were collected in the 4000–650 cm^−1^ domain, on a diamond/ZnSe UATR device. Spectra were built up after a number of 64 co-added scans, with a resolution of 2 cm^−1^. The spectral domain 2300–1900 cm^−1^ has no spectroscopic significance; the existing bands being represented by the noise signal of the ATR crystal.

### 2.4. Thermal Stability Investigations

Thermal analysis investigations were carried out on a Perkin-Elmer DIAMOND apparatus (Perkin-Elmer Applied Biosystems, Foster City, CA, USA) for simultaneously obtaining the TG (thermogravimetric/mass curve), DTG (derivative thermogravimetric/mass derivative curve) and HF (heat flow), in dynamic air atmosphere (100 mL·min^−1^), using open aluminum crucibles. The analyses were carried out under non-isothermal conditions at a heating rate β = 10 °C·min^−1^ from ambient up to 400/500 °C. For determining the thermal effects, the DTA data (in µV) were converted in HF data (mW).

In order to assure the reproducibility of TG study, each analysis was repeated three times and the results were practically identical.

## 3. Results and Discussion

The goal of this study was to obtain information regarding the stability and compatibility of LTSS with several pharmaceutical excipients, under ambient conditions and, later, under thermal stress. Since in the case of thyroid hormone replacement therapy, the amount of API administered daily to patients that underwent total thyroidectomy is usually below 200 μg [32], and the common mass of the tablet is 100 mg (consisting mainly in excipients, while API content is usually between 25 and 300 μg [5]), the drug–excipient interactions are likely to be clinically significant given the low dose of the API in this type of formulation. In other words, if interactions can be detected at relatively high drug concentrations as in binary mixtures, certainly they take place even in actual formulations, where the quantity of excipients is even higher, leading to improper formulations in terms of bioavailability.

As current practice in preformulation studies of solid or semisolid dosage forms, the analysis of binary mixtures consisting of equal masses of API and pharmaceutical excipients were also reported for antiviral drug penciclovir [33], ketoconazole [34], atorvastatin calcium [35], rivaroxaban [36], roflumilast [37] or diazepam [38]. Initially, the binary mixtures were subjected to UATR-FTIR analysis in order to evaluate the interactions that may occur under ambient conditions, and later to thermal stress, in order to get an in-depth view for the thermal-induced interactions.

### 3.1. UATR-FTIR Analysis

**Figure 2:**
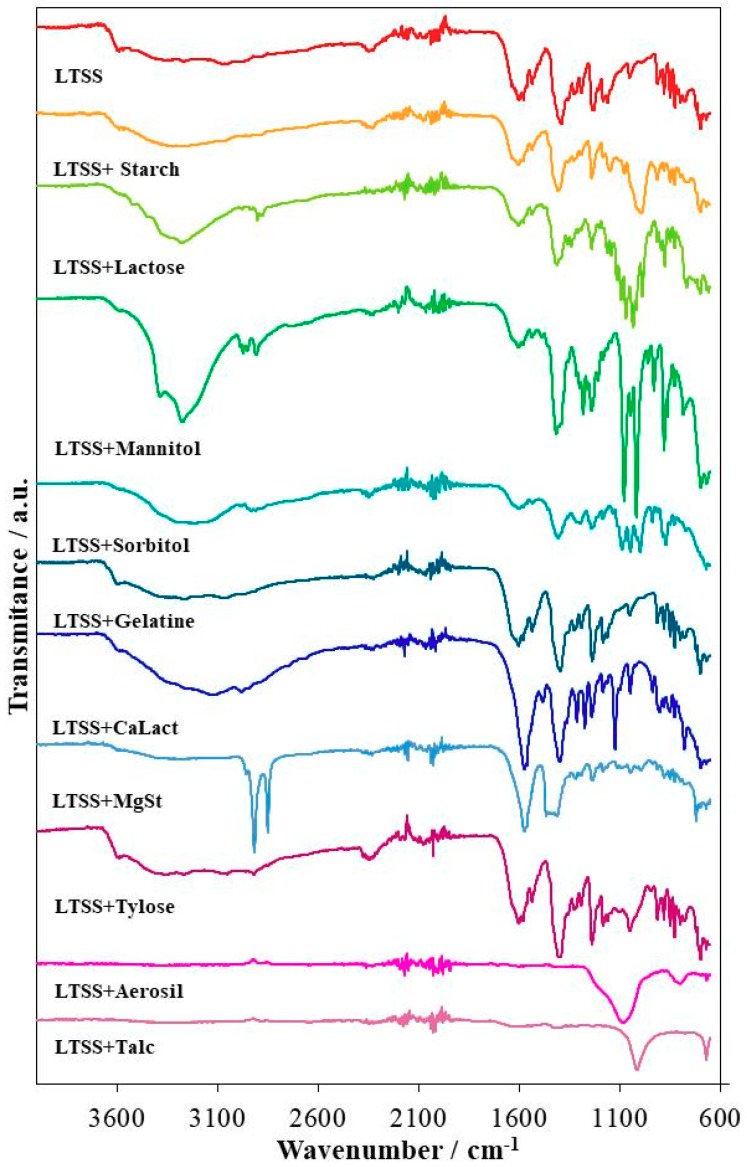
Universal attenuated total reflectance-Fourier transform infrared spectroscopy (UATR-FTIR) spectra of LTSS and binary mixtures with excipients.

As to bring to light the possible interactions between the active substance LTSS and each selected excipient, the FTIR analysis was performed, the obtained spectra being revealed in Figure 2. As previously reported by our research team, the spectra of pure LTSS revealed the presence of bands associated with the most important structural characteristics [15]. The stretching vibration of the O−H bond from the water that is found in the crystalline framework determined the presence of the broad spectral band seen in the 3650–2750 cm^−1^ range. Although mostly overlapped by the O−H band, the stretching vibration of the C−H bond from the methylene group, as well as from the aromatic rings can still be observed at 2939, 2868 and 3053 cm^−1^, respectively. The stretching vibration of the phenolic O−H bond can be seen at 3589 cm^−1^, while the same vibration type associated with the N−H bonds determine the presence of multiple bands in the 3200–3500 cm^−1^ spectral range that are overlapped, for the most part, by the O−H band. The bending vibration of the latter bonds can be associated with the peak seen at 1630 cm^−1^, while the stretching vibration of the C−N bond is revealed by the band observed at 1053 cm^−1^. The stretching vibration of the C=C bonds from the aromatic systems can be correlated with the bands seen at 1455, 1500 and 1536 cm^−1^, whereas the bending vibrations of the aromatic C−H bonds are revealed by peaks observed at 1162 (in-plane deformation) and 913 and 881 cm^−1^ (out-of-plane deformation). At 1578 and 1390 cm^−1^ the asymmetric and, respectively, the symmetric stretching vibration of the C=O bond from the carboxylate anion are revealed, while the vibration of the C−O bond is marked by the appearance of a band at 1183 cm^−1^ on the spectra of LTSS.

Regarding the FTIR spectra and obtained data (Table 1) for each binary mixture containing LTSS, it can be said that although slight band shifts and intensity decrease were observed, the most similar results to those seen for pure LTSS were revealed for the LTSS+Starch, LTSS+Gelatin and LTSS+Tylose mixtures. Easily visible spectral differences were observed for the mixtures LTSS+Lactose, LTSS+Mannitol and LTSS+Sorbitol, for which the band associated with the vibration of the O−H bond was modified, presenting with increased intensity due to the large number of hydroxyl moieties of each excipient. Since each of these excipients presented with numerous aliphatic hydrocarbon moieties, the stretching vibration of the C−H bonds are more easily observed. A few bands of pure LTSS shifted or were overlapped by some of the bands associated with the structural characteristics of each excipient and in the case of LTSS+Lactose a series of new bands were revealed, these facts indicating the probability of an interaction occurring in the lastly presented three mixtures. Band shifts or disappearance were also observed for the mixtures LTSS+CaLact and LTSS+MgSt suggesting possible interactions occurring between the active substance and each excipient in solid state, although their spectra maintained some of the characteristics associated with pure LTSS. For these two excipients (CaLact and MgSt), solely the thermal analysis can indicate the compatibility or incompatibility with LTSS. This was not the case for the mixtures LTSS+Aerosil and LTSS+Talc, for which the broad, intense band correlated with the stretching vibration of the Si-O bond seen at 1081 and 1013 cm^−1^, respectively, determined the severe modification of the initial LTSS spectra, almost all its peaks being unobservable.

### 3.2. Thermal Stability Investigations

**Figure 3:**
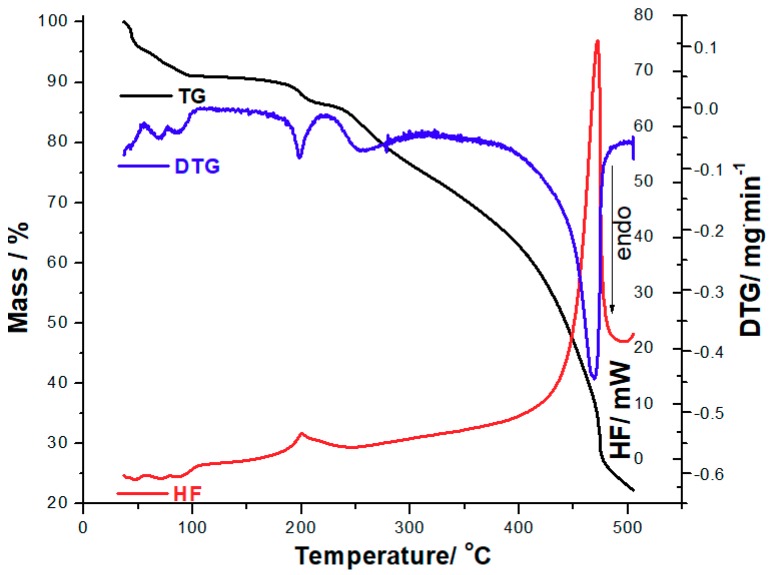
Thermoanalytical curves (thermogravimetric TG/ derivative thermogravimetric DTG/heat flow HF) obtained at β = 10 °C·min^−1^ in dynamic air atmosphere for LTSS.

LTSS presents a complex pathway of decomposition, as suggested by the thermoanalytical curves obtained at β = 10 °C·min^−1^ presented in Figure 3. The complex thermoanalytical profile is due to the presence of water that is found in the crystalline framework of the sodium salt of levothyroxine. Even if the supplier of LTSS indicate that the compound is a hydrate, without a clear information of the water content, in our previous paper we have established the salt is a pentahydrate [15], which is also confirmed by the current analysis. The mass loss Δ*m* = 9.48% that takes place between 35 and 103 °C is represented by release of water found in the crystalline framework, corresponding to the water content of 5 mol per mol of salt. Anhydrous LTSS is thermally stable up to 156 °C, when a decomposition process begins, up to 500 °C, with a total mass loss Δ*m* = 76.95%. Separation of individual processes regarding the thermodegradation of LTSS is not possible to be realized based on TG curves, since the mass loss is continuous with the advance of temperature, except the dehydration step, with the formation of stable anhydrous LTSS.

The separation of degradation steps that took place during thermolysis of LTSS is revealed by the DTG curve, as follows: dehydration is represented by two enchained processes, namely first process (I) in the 35–77 °C temperature range (DTG_peak_ at 69 °C, Δ*m*_1_ = 6.85%); second process in the 77–103 °C temperature range (DTG_peak_ at 87 °C, Δ*m*_2_ = 2.63%); in the 103–156 °C temperature range, anhydrous LTSS is stable and no mass loss occur; third degradative process takes place between 156 and 220 °C, with DTG_peak_ at 198 °C and a corresponding mass loss Δ*m*_3_ = 4.22%; fourth process takes place between 220 and 284 °C, with DTG_peak_ at 254 °C and a corresponding mass loss Δ*m*_4_ = 22.05%; the last process (the fifth) takes place between 284 and 500 °C, with DTG_peak_ at 469 °C and a corresponding mass loss Δ*m*_5_ = 41.20%. Following these considerations, it can be summed up that LTSS is almost completely degraded at 500 °C, when the residual mass is 23.05%.

The HF curve reveals endothermal removal of water molecules also in the 35–103 °C temperature range, in good agreement with the DTG curve, with HF_max_ at 47, 72 and 92 °C, followed by an exothermal event between 182 and 240 °C, with a HF_max_ = 201 °C and Δ*H* = −95.2 J g^−1^, in agreement with previously published results [15]. Also, in the 405–500 °C temperature range, an intense exothermal event is observed, associated with the advances thermolysis of organic skeleton of levothyroxine (HF_max_ = 472 °C). It worth mentioning the exothermal event observed on the HF curve that has a peak at 201 °C—the first degradative process due to thermooxidations of LTSS. Due to its saline structure, it was expected that a phase transition (melting) cannot occur for LTSS. So, in the compatibility study of LTSS with excipients, the disappearance of this peak will suggest the occurring thermal-induced interactions of the API and coformulation compound.

**Figure 4:**
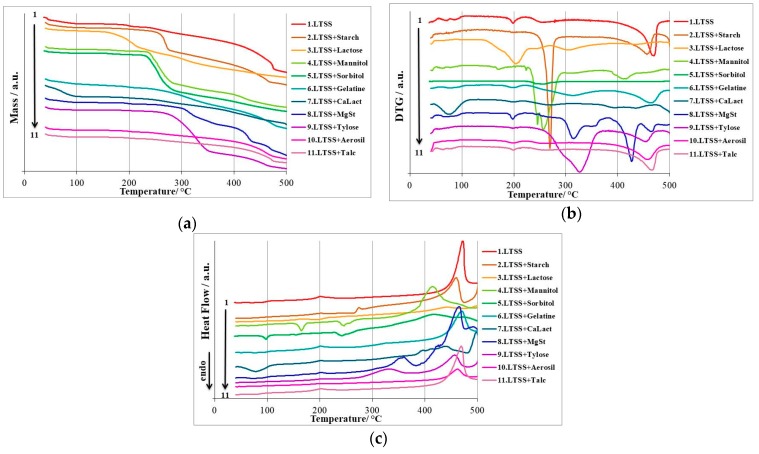
Superimposed thermoanalytical curves registered for LTSS and binary mixtures with excipients: (**a**) Mass vs. temperature curves (TG curves); (**b**) Mass derivative vs. temperature curves (DTG curves); (**c**) Heat flow vs. temperature curves (HF curves).

The superimposed thermoanalytical curves registered for LTSS and binary mixtures with excipients, namely, mass vs. temperature (TG), mass derivative vs. temperature (DTG) and heat flow vs. temperature (HF) curves are presented in Figure 4.

The thermoanalytical curves determined from the LTSS+Starch binary mixture reveal a two-step decomposition process, according to the DTG curves. Since the mass loss is continuous during thermal treatment of the sample, the formation of a stable intermediate was not evidenced. The mass loss that takes place in the 35–120 °C is due to dehydration of both LTSS and starch, but these processes are not well individualized on DTG and HF curves, probably due to “linear” release of water from the mixture. The main decomposition process of the mixture takes place in the 227–508 °C temperature range, with a considerable mass loss (Δ*m* = 78.52%, so the residual mass is 10.21%. the mass loss is rapid between 239 and 281 °C, with a maximum at 270 °C. The HF curve reveals the exothermic decomposition of LTSS in the 186–218 °C temperature range, with HF_max_ at 201 °C, as in the case of pure LTSS, suggesting that no interaction take place up to this temperature. Other intense exothermic events are observed at higher temperatures, due to thermolysis of API and carbohydrate skeleton of starch (peaks at 274, 459 and 504 °C).

In the case of LTSS+Lactose mixture, the dehydration that takes place in the 40–105 °C temperature range is due to dehydration of LTSS (since the mass loss is half the values corresponding to pure LTSS), also confirmed by the HF curve, by the process that took place between 40 and 49 °C, with an endothermic HF_peak_ at 43 °C. DTG curves reveal a considerable mass loss in the 125–505 °C interval, with intense peaks at 203, 305 and 435 °C. However, it seems that the thermal degradation of lactose is overlapped with the decomposition of LTSS that should appear at 201 °C, which is no longer present on the curve. In this case, an interaction between LTSS and lactose is suspected.

The mixture of LTSS+Mannitol show a mass loss of 3.96% up to 89 °C, due to dehydration of LTSS, process also confirmed by the DTG curves and HF, in the 40–93 °C temperature range, with peaks at 43 and 62 °C, respectively. First step of decomposition of Mannitol, along with its melting, took place between 163 and 187 °C, as suggested by all thermoanalytical curves, with a well-defined event on the HF curve, between 151 and 178 °C and a HF_peak_ at 165 °C. The decomposition peak of LTSS is no longer revealed by the HF curve, suggesting the thermal-induced interaction between the components of the binary system. With the increase of temperature, the degradation of the components advances, so the most rapid mass loss takes place between 224 and 285 °C, as suggested by all thermoanalytical curves. It is interesting to notice the endothermic event that is revealed by the HF curve, suggesting probably the condensation of melted mannitol molecules, with release of water molecules, and later the thermooxidation of the hydrocarbonated skeleton, at temperature over 339 °C.

Since the structural similarity between Sorbitol and Mannitol, the thermal profile of binary mixture LTSS+Sorbitol is very similar to the one previously discussed. Sorbitol is an isomer of mannitol, the two differ only in the orientation of the hydroxyl group on the C2. At temperature below 83 °C, the dehydration of sample occurs, by the loss of crystallization water from LTSS. The melting of Sorbitol takes place between 83 and 106 °C, with a HF_peak_ at 97 °C. In this case too, due to the melting of Sorbitol, the decomposition of LTSS is no longer visible, suggesting a possible interaction between the components. The behavior of mixture at temperatures higher than 217 °C is similar to the one containing Mannitol.

Gelatin is compatible with LTSS, since the water loss and first decomposition of the API is present on thermoanalytical data of the binary mixture. TG data suggest a continuous mass loss, without formation of stable intermediates. Four decomposition steps are suggested by both DTG and HF curves, the later showing endothermic peaks due to dehydration similar to LTSS, at 48, 75 and 91 °C, respectively. Intense thermooxidations occur in the range 412–500 °C, with a total mass loss of 70.15%.

The thermoanalytical data regarding the stability of the LTSS+CaLact mixture reveal a rapid mass loss up to 115 °C (Δ*m* = 16.07%), due to overlapped dehydration of pentahydrate excipient and pentahydrate API. The dehydration is also confirmed by the DTG curve, with a process taking place between 45 and 117 °C, with DTG_max_ at 75 °C, and a well-defined and broad endothermic event on the HF curve in the 47–110 °C range, with peak at 78 °C. The formation of anhydrous mixture between LTSS and CaLact is revealed by the TG/DTG curves in the 115–165 °C temperature range, the mass loss being around 0.5% in this interval. In the solid anhydrous mixture of both salts (API and excipient), the first decomposition process occurs for LTSS, as suggested by the HF curve, by the exothermic process occurring between 192 and 214 °C, with a HF_peak_ at 202 °C. With the advance of the thermal treatment of the sample, the degradation of excipient occurs, the events being overlapped with the degradation of LTSS. The residual mass is considerable (42.89%), due to the formation of some chars containing Ca^2+^ and Na^+^ cations. This behavior of LTSS in binary mixture with CaLact suggest that this excipient can be used in the development of new generic formulations.

It is well known that magnesium stearate (MgSt) is an excipient that commonly shows interactions to various APIs [25,39]. Surprisingly, in the case of the LTSS+MgSt mixture, interaction is not observed. The pathway of degradation is complex: the dehydration is sustained by the mass loss in the 40–100 °C range, TG curves revealing Δ*m* = 6.06%. The HF curve reveals the decomposition of LTSS in the 192–215 °C range, with HF_max_ at 201 °C, as in the case of pure LTSS.

The mixture LTSS+Tylose has a good thermal stability and no interactions are suggested. The dehydration of API is complete at 82 °C, with a corresponding mass loss Δ*m* = 4.38%. Anhydrous LTSS is compatible with Tylose, and the mass is almost constant in the 82–187 °C temperature range. At 192 °C, the decomposition of LTSS begins, with a maximum on the HF curve at 202 °C and the end of the process at 214 °C. At temperature higher than 227 °C, DTG curves reveal a rapid mass loss (DTG_max_ at 326 °C, Δ*m* = 58.81%.) up to 370 °C, due to oxidative thermolysis of LTSS and Tylose (exothermic event suggested by the HF curve in the range 281–375 °C, peak at 334 °C).

The mixture of LTSS with the inorganic excipient SiO_2_ (Aerosil) show compatibility, since the thermal events observed for the mixture are due to the presence of LTSS. Dehydration is complete at 86 °C, and the mass loss observed in this range is Δ*m* = 4.47%, confirming the fact that the excipient is not hydrated. The HF curve shows the characteristic decomposition interval of LTSS, in the 192–212 °C range, with a maximum at 201 °C. The residual mass is considerable (54.46%), since inorganic excipient remains in the char, with unaltered structure.

Similar discussion can be carried out for the mixture LTSS+Talc, another thermal inert excipient. Dehydration of LTSS is revealed by all thermoanalytical curves, as follows: TG curves indicate a mass loss Δ*m* = 4.30% (approximately half the value observed for pure LTSS) up to 90 °C. Also, the HF curve reveals three endothermic events in this temperature interval, with peaks at 44, 64 and 79 °C, respectively. Also, the HF curve reveals the decomposition of LTSS between 186 and 231 °C, with a maximum at 201 °C. Corroborating the data from thermoanalytical curves of LTSS+Talc mixture, it can be concluded that no thermal-induced interactions occur and this versatile excipient generally used as glidant can be incorporated in new generic formulations containing sodium levothyroxine.

The summarization of thermoanalytical data is presented in Table 2.

In previous papers, we also carried out diffractometric (XRPD) studies on binary mixtures kept under ambient conditions, when the corroboration of thermoanalytical data with the ones suggested by the FTIR spectroscopy were not concluding [23,25,26,35,39]. However, in the case of LTSS mixtures with selected excipients, no discrepancies were noticed between the selected instrumental techniques, as the incompatibilities suggested by the FTIR study were confirmed by the thermal analysis.

## 4. Conclusions

This study presents a preformulation study for the synthetic hormone levothyroxine (namely, the pentahydrate sodium salt, abbreviated as LTSS), used as an active pharmaceutical ingredient in both original and generic formulations. Since the amount of levothyroxine in solid formulations (tablets) is considerably lower in comparison to the amount of excipients (even 1000 fold lower), the interactions that could occur can lead to a drastic diminution of therapeutic activity, i.e., bioavailability. As selected excipients, the following were studied: starch, anhydrous lactose, D-mannitol, D-sorbitol, gelatin, calcium lactate pentahydrate, magnesium stearate, methyl 2-hydroxyethyl cellulose (Tylose), colloidal SiO_2_ (Aerosil) and talc. The binary mixtures LTSS+Excipient were prepared by trituration of components in a 1:1 (mass/mass) ratio in order to accentuate the drug–excipient interaction that may occur under ambient conditions and under thermal stress. As investigational tools, UATR-FTIR spectroscopy and thermal analysis were chosen and used as follows: UATR-FTIR spectra were drawn up for samples kept under ambient conditions, while thermoanalytical tools (TG/DTG/HF data) were chosen to evaluate the appearance of interactions during thermal treatment. Synthetically, it can be said that LTSS is incompatible with lactose, mannitol and sorbitol, as suggested by both spectroscopic and thermal results, and should be avoided in the development of new generic formulations.

## Figures and Tables

**Table 1:** Universal attenuated total reflectance-Fourier transform infrared spectroscopy) (UATR-FTIR) spectral bands obtained for LTSS and each binary mixture.

Sample	Characteristic UATR-FTIR Bands/cm^−1^
LTSS	3650–2750 (wide band); 3589; 3568; 3526; 3448; 3266; 3052; 2939; 2868; 1630; 1603; 1578; 1536; 1500; 1455; 1390; 1327; 1291; 1235; 1183; 1162; 1053; 913; 881; 849; 827; 799; 774; 712; 702; 698; 669
LTSS+Starch	3650–2750 (wide band); 3589; 3568; 3525; 3447; 3051; 2940; 2879; 1631; 1603; 1581; 1535; 1451; 1406; 1329; 1293; 1239; 1183; 1147; 993; 914; 881; 849; 829; 800; 767; 715; 708; 699; 669
LTSS+Lactose	3589; 3550–3000 (wide band); 3522; 3448; 3278; 3052; 2977; 2932; 2901; 2876; 1637; 1604; 1579; 1536; 1476; 1449; 1412; 1359; 1341; 1308; 1293; 1241; 1202; 1161; 1142; 1116; 1094; 1070; 1033; 1019; 988; 949; 915; 892; 877; 849; 828; 768; 721; 700; 668
LTSS+Mannitol	3588; 3500–3000 (wide band); 3385; 3275; 2986; 2972; 2851; 2906; 1603; 1580; 1536; 1488; 1417; 1356; 1314; 1301; 1282; 1240; 1209; 1184; 1078; 1046; 1019; 960; 930; 881; 864; 828; 784; 698; 668
LTSS+Sorbitol	3588; 3500–3000 (wide band); 2985; 2948; 2931; 2909; 2876; 1602; 1578; 1534; 1458; 1408; 1315; 1295; 1238; 1184; 1133; 1089; 1046; 1015; 999; 938; 914; 883; 872; 849; 828; 769; 669
LTSS+Gelatin	3589; 3550–3850 (wide band); 3271; 3050; 2917; 2848; 1603; 1579; 1535; 1395; 1328; 1291; 1238; 1183; 1163; 1053; 914; 881; 849; 828; 812; 800; 776; 722; 709; 699; 669
LTSS+CaLact	3589; 3550–2700 (wide band); 3130; 2979; 2748; 2657; 1571; 1483; 1400; 1313; 1274; 1240; 1184; 1124; 1048; 937; 903; 882; 850; 828; 779; 721; 703; 698; 669
LTSS+MgSt	3577; 3500–3050 (wide band); 2955; 2917; 2850; 1571; 1466; 1449; 1433; 1411; 1318; 1291; 1237; 1184; 1114; 1044; 994; 914; 881; 849; 828; 799; 721; 698; 669
LTSS+Tylose	3589; 3530–2800 (wide band); 3269; 3048; 2919; 2850; 1603; 1579; 1536; 1396; 1328; 1292; 1239; 1183; 1162; 1052; 914; 881; 849; 828; 800; 776; 721; 709; 699; 669
LTSS+Aerosil	1250–990 (broad band—peak:1081); 799; 668
LTSS+Talc	3580–3180 (wide band); 1630; 1412; 1237; 1080–915 (broad band—peak:1013); 670

**Table 2:** Summarization of thermoanalytical data (TG/DTG/HF) obtained after thermal analysis of binary mixtures.

Sample	Step	TG		Δm/%	DTG			HF		
		T_onset_/°C (m% at T_onset_)	T_offset_/°C (m% at T_offset_)		T_onset_/°C	T_offset_/°C	T_peak_/°C	T_onset_/°C	T_offset_/°C	T_peak_/°C
LTSS	I+II	35 (100%)	102 (90.52%)	9.48	35 77	77 103	69 87	35 54	54 103	47 72;92
	III	160 (90.52%)	223 (86.30%)	4.22	156	220	198	182	240	201
	IV+V	223 (86.30%)	500 (23.05%)	63.25	220 284	284 500	254 469	405	500	472
LTSS+Starch	I	35 (100%)	227 (88.73%)	11.27	-	-	-	186 258 389 475	218 280 475 508	201 274 459 504
	II-IV	227 (88.73%)	508 (10.21%)	78.52	240 390 485	285 471 508	270 456 503			
LTSS+Lactose	I	40 (99.69%)	105 (95.97%)	4.03	49	83	61	40 152 413	49 191 470	43 166 444
	II-IV	125 (95.45%)	505 (30.45%)	65	143 270 403	230 389 470	203 305 435			
LTSS+Mannitol	I	40 (99.67%)	89 (96.04%)	3.96	40	93	43;62	40 151 234 385	93 178 270 486	43; 62 165 246 414
	II	136 (95.78%)	200 (93.69%)	2.09	157	186	170			
	III-V	224 (93.14%)	504 (14.75%)	78.39	227 248 378	248 293 439	245 256 412			
LTSS+Sorbitol	I	37 (100%)	83 (97.02%)	2.98	37	47	41	37 83 227 322 454	48 106 278 454 505	43 97 241 417 471
	II	151 (96.64%)	187 (95.08%)	1.56	144	177	156			
	III	217 (94.40%)	505 (15.07%)	79.33	227	298	255			
LTSS+Gelatin	I-IV	40 (100%)	505 (29.85%)	70.15	59 167 221 382	110 221 382 505	77 200 315 463	40 186 412	108 230 500	48; 75; 91 201 470
LTSS+CaLact	I	40 (99.61%)	115 (83.93%)	16.07	45	117	75	47 192 383 403 412 480	110 214 403 421 480 503	78 202 395 414 440 489
	II-V	165 (83.42%)	508 (42.89%)	40.53	160 216 364 412	216 313 412 470	199 268 394 435			
LTSS+MgSt	I	40 (99.68%)	100 (93.94%)	6.06	53	99	72	192 325 398 427 476	215 383 427 476 509	201 360 426 465 492
	II	177 (93.30%)	210 (91.65%)	1.65	187	220	198			
	III-VI	237 (91.27%)	509 (17.59%)	73.68	294 339 398 444	340 358 445 476	315 350 427 465			
LTSS+Tylose	I	40 (99.57%)	82 (95.62%)	4.38	-	-	-	192 281 375	214 375 480	202 334 457
	II-III	187 (94.91%)	508 (12.09%)	82.82	227 385	370 477	326 452			
LTSS+Aerosil	I	40 (99.55%)	86 (95.53%)	4.47	-	-	-	40 192 418	46 212 462	43 201 491
	II-IV	152 (95.00%)	504 (54.46%)	40.54	171 222 384	222 295 495	200 257 458			
LTSS+Talc	I	40 (99.72%)	90 (95.70%)	4.30	51	72	65	40 186 406	90 231 504	44; 64; 79 201 469
	II-III	172 (95.15%)	504 (59.38%)	35.77	176 369	216 488	199 466			
